# Catalytic Activity of Silicon Nanowires Decorated with Gold and Copper Nanoparticles Deposited by Pulsed Laser Ablation

**DOI:** 10.3390/nano8020078

**Published:** 2018-01-30

**Authors:** Michele Casiello, Rosaria Anna Picca, Caterina Fusco, Lucia D’Accolti, Antonio Alessio Leonardi, Maria Josè Lo Faro, Alessia Irrera, Sebastiano Trusso, Pietro Cotugno, Maria Chiara Sportelli, Nicola Cioffi, Angelo Nacci

**Affiliations:** 1 Dipartimento di Chimica, Università di Bari, Via E. Orabona, 4, 70126 Bari, Italy; michele.casiello@uniba.it (M.C.); rosaria.picca@uniba.it (R.A.P.); lucia.daccolti@uniba.it (L.D.); pietro.cotugno@uniba.it (P.C.); maria.sportelli@uniba.it (M.C.S.); 2 CNR-ICCOM, UOS Bari, Via E. Orabona, 4, 70126 Bari, Italy; fusco@ba.iccom.cnr.it; 3 CNR IPCF, Viale Ferdinando Stagno d’Alcontres, 37, 98158 Messina, Italy; antonio.leonardi@ct.infn.it (A.A.L.); mjolofaro@gmail.com (M.J.L.F.); irrera@me.cnr.it (A.I.); trusso@me.cnr.it (S.T.); 4 Dipartimento di Fisica ed Astronomia, Università di Catania and INFN Sezione di Catania, Via Santa Sofia, 68, 95125 Catania, Italy

**Keywords:** C_aryl_–N coupling, reduction of nitroarenes, Si nanowires, Au nanoparticles, Cu nanoparticles

## Abstract

Silicon nanowires (SiNWs) decorated by pulsed laser ablation with gold or copper nanoparticles (labeled as AuNPs@SiNWs and CuNPs@SiNWs) were investigated for their catalytic properties. Results demonstrated high catalytic performances in the C_aryl_–N couplings and subsequent carbonylations for gold and copper catalysts, respectively, that have no precedents in the literature. The excellent activity, attested by the very high turn over number (TON) values, was due both to the uniform coverage along the NW length and to the absence of the chemical shell surrounding the metal nanoparticles (MeNPs). A high recyclability was also observed and can be ascribed to the strong covalent interaction at the Me–Si interface by virtue of metal “silicides” formation.

## 1. Introduction

Silicon nanowires (SiNWs) are attracting worldwide research interest due to their fascinating properties and application in a wide range of areas, such as electronics, photovoltaics, solar cells, diagnostics, molecular sensing, batteries, catalysis, and analytical chemistry [1,2,3,4,5,6,7].

Several methods have been employed to fabricate SiNWs such as vapor-liquid-solid growth [8,9], chemical vapor deposition [10], laser ablation [11], thermal evaporation [12], metal-assisted chemical etching (MACE) [13,14], etc. Among them, the chemical etching approach proved to be a more rapid and practical technique for producing uniform SiNWs at room temperature with inexpensive and scalable procedures [15,16,17].

SiNWs are also a promising host matrix for dispersing metal nanoparticles (MeNPs) due to their large surface area, mechanical stability, low cost, and easy preparation by industrially compatible methods [18]. The SiNW-dense forest structure offers a wide range of possibilities for further implementations. 

For the decoration of SiNWs with MNPs, chemical routes are the most adopted approaches, even if they do not allow for fine control of the NP growth, size, and shape. In addition, with galvanic reduction metal clusters are often surrounded by a chemical shell that limits the applications of such systems. A further concern is the incomplete NW coverage that very often occurs only on the top of the NW arrays, thus limiting the performance of the nanocomposite [19,20,21].

In the last decade, several nanoscale metals have been anchored on SiNWs for catalytic purposes (Au [22,23], Ag [24,25,26], Pd [27,28,29,30], Pt [31,32], Cu [33,34] etc.). Decoration by chemical reduction has been frequently employed, with applications mainly in the degradation of dyes and pollutants [22,24,25,26,27,28,29,30,31,33], but also in cross-coupling reactions [3,27,28,34].

Recently, a physical method based on the pulsed laser ablation technique was successfully used for deposition of metal nanoparticles on substrates with different surface morphologies [35,36,37]. By selecting the proper values of experimental parameters (e.g., laser fluence, gas nature, and pressure, number of laser pulses etc.), a uniform and dense decoration of silicon nanowires was obtained with a tight control of size, distribution, and mutual distance among NPs. In the case of silver NPs, this material has been successfully used for Surface Enhanced Raman Spectroscopy (SERS) applications [36,37,38].

Although SiNWs hybrid composites had already been investigated in various fields, none of those prepared via laser ablation have been tested in catalysis. On these bases, above all due to the absence of the chemical shell surrounding NPs, we envisaged that these materials should display superior catalytic properties. 

In the present work, we report on the synthesis and characterization of gold and copper-based nanocomposite systems (namely AuNPs@SiNWs and CuNPs@SiNWs) and their use as highly recyclable heterogeneous catalysts. Specific chemical transformations were chosen as a benchmark for these materials, ranging from the well-known reductions of nitroarenes [22,23,33] to the relatively unexplored C–N hetero-couplings of aryl halides [34]. Concerning the latter processes, we found, unprecedented in the literature, that gold catalyst (AuNPs@SiNWs) can efficiently promote C_aryl_–N coupling with amines. We also found that the analogous copper-based system (CuNPs@SiNWs) is an active catalyst for alkoxy- and amino-carbonylation of haloarenes.

## 2. Materials and Methods

### 2.1. Materials

Aryl halides, amines, alcohols, nitroarenes, Cesium carbonate, Potassium carbonate, Triethylamine, Tetrabutylammonium acetate, bidistilled water and Sodium tetraborohydride were purchased from Sigma Aldrich and used as received. Solvents CH_3_CN, DMF, DMSO, THF, DMA, Et_2_O (Sigma Aldrich, Milan, Italy) were dried prior to use.

Preparation of silicon nanowires and their decoration with gold and copper nanoparticles by PLD technique were accomplished by previously reported protocols [38], and are briefly described into the paragraph on the structural properties of catalysts nanocomposites (results and discussion section).

Reaction products were detected by Gas chromatography-mass spectrometry (GC-MS) and identified by comparison of their MS spectra with the literature data. GC-MS spectra were recorded on a Shimadzu GLC 17-A gas-chromatograph connected with a Shimadzu GLC/MS QP5050A selective mass detector (capillary column: HP-5 MS, 30 m).

XPS analyses of silicon nanocomposites were performed using a PHI Versaprobe II spectrometer equipped with a monochromatized Al Kα source (1486.6 eV). Dual-beam charge neutralization was constantly applied during analysis. Large area XPS was performed operating with a sampling area of 200 × 1400 µm^2^. Survey and high-resolution (HR) spectra were acquired at a pass energy value of 117.4 and 58.7 eV, and energy step of 1.0 and 0.125 eV, respectively. HR regions relevant to C1s, O1s, Au4f (for AuNP-modified materials), Cu2p_3/2_ (for CuNP-modified materials), Si2p, N1s (after reactions), and CuL_3_M_45_M_45_ were acquired. Multipak software (v.9.7.0.1, Ulvac-Phi Inc., Chigasaki, Japan) was used for elemental quantification. CasaXPS^®^ software (v. 2.3.18PR1.0, Casa Software Ltd., Teignmouth, UK) was used for fitting XP spectra. Binding Energy (BE) was referred to aliphatic component of C1s at 284.8 eV.

### 2.2. General Procedure for C_aryl_–N Cross Coupling

All the reactions were carried out in a sealed glass vial. In a typical procedure, in a 10 mL glass vial equipped with a magnetic bar, haloarene (0.25 mmol), amine (2.5 mmol), anisole (or 4-methylanisole as internal standards) and Cs_2_CO_3_ (1 mmol) were suspended in 2.0 mL of bidistilled water. Then, the square silicon wafer of MeNPs@SiNWs catalyst (1.5 µm, 1 cm^2^, Me = Au or Cu) was immersed into the liquid and heating and stirring were started keeping the reaction mixture at 110 °C for 8 h. After reaction time, the silicon sheet was recovered with tweezers and washed three times with bidistilled water, then with acetone. Next, after drying with a N_2_ stream, silicon wafer was reused for a new run or stored in a glass vial under inert atmosphere. The reacted mixture was extracted with ethyl acetate, and the organic phase analyzed by GC-MS for evaluating conversions and selectivities listed in Table 1.

### 2.3. General Procedure for Carbonylation of Haloarenes

All the carbonylative couplings were carried out in a stainless-steel autoclave equipped with a glass vial to avoid the direct contact of the reaction mixture with the metal wall. In a typical procedure: a glass vial equipped with a magnetic bar was charged with haloarene (0.25 mmol), amine (0.625 mmol), Cs_2_CO_3_ (1 mmol), and anisole (or 4-methylanisole as internal standards) suspended into 2 mL of bidistilled water. A square silicon wafer of CuNP@SiNWs (1.5 µm, 1 cm^2^) was then immersed into the reaction mixture and the vial inserted into the autoclave. Once sealed, the reactor was filled with carbon monoxide at 20 atm and heated under stirring at 110 °C for 8 h. After reaction time, the residual gas was discharged, the silicon sheet was recovered with tweezers and the reaction mixture extracted with ethyl acetate for GC-MS analyses.

A similar procedure was employed for alkoxy carbonylation, providing that the alcoholic reagent was used as a reaction medium in place of water (2 mL), heating was raised at 130 °C, under P_CO_ = 30 atm and by adding Et_3_N (1 mmol) as an additive.

Reaction products (benzamides and benzoates) listed in Table 2 and Table 3 were identified by comparison of their MS spectral data with those reported in the literature (see supplementary materials).

### 2.4. General Procedure for Reduction of Nitroarenes

All reactions were carried out in a sealed glass vial. Firstly, a series of concentrated mother solutions of nitroarenes were prepared. With a micropipette some microliters of these concentrated solutions were added to a reductant agent solution and filled with water until to obtain the selected concentration. Finally, a square silicon wafer containing gold NPs or copper NPs was immersed in the reaction mixture. After reaction time, the silicon sheet was recovered with tweezers and washed three times with bidistilled water, then with acetone and after drying with a N_2_ flux, stored in a glass vial in an inert atmosphere for further catalytic cycle. The reacted mixture was analyzed directly or by extraction with ethyl acetate, by GC-MS instrument. The MS data are reported in supplementary materials.

## 3. Results and Discussion

### 3.1. Structural Properties of Catalysts Nanocomposites

SiNWs are grown on 100 oriented crystalline p-type Si substrates by silver salts metal-assisted chemical etching. An oxygen-free surface is obtained processing the samples with 2 min of UV ozone cleaning and 5 min of chemical etching in a 5% hydrofluoric (HF) water solution. The clean substrates are immersed in the etching solution made of AgNO_3_ 40%, H_2_O 40% and HF 20%. Each step of SiNW growth is performed at room temperature.

During the process, silver salts dissolve in the solution leading to the formation and precipitation of small Ag clusters that act as precursors for the SiNW synthesis. Due to the difference of electronegativity at the Ag–Si interface an anodic reaction occurs, producing a thin layer of silicon dioxide only underneath the metal covered regions that is selectively etched by HF, causing the metal nanoparticle to sink into the Si bulk layer to form NWs.

1.5 µm long Si nanowires were prepared, depending on the etching conditions and time. Decoration of the SiNWs was realized by pulsed laser deposition (PLD) of gold or copper nanoparticles. The process is carried out in a high vacuum chamber with a residual base pressure of 10^−4^ Pa. The laser beam from a KrF excimer laser (Lambda Physik CompEx 205, 25 ns pulse width, 248 nm wavelength, 10 Hz repetition rate, laser fluence set at 2.0 J·cm^−2^) is focused onto the surface of a pure gold or copper target using a quartz lens with the target mounted on a rotating holder to avoid excessive surface damaging. 

SiNW samples are positioned 35 mm far from the target surface and the Au deposition process is performed in presence of 100 Pa of Ar, while, for Cu deposition, Ar pressure is set at 70 Pa. The difference in the ambient gas pressure for the two metals is due to the different atomic mass values (Cu 63.5 amu, Au 196.9 amu). The NPs formation mechanism is, in fact, driven by the interaction of the laser generated plasma with the ambient gas [39]. As a consequence, a higher Ar pressure is needed to confine the Au plasma with respect to the Cu one.

Under the selected deposition conditions, the plasma expands through the ambient gas forming a shock wave [40], at the interface between the plasma and the atmosphere ambient. Gas density and pressure favor the in-flight formation of clusters that subsequently land onto the substrate surfaces. Deposition are performed using a laser pulse number of 60,000 to realize decorated SiNWs.

**Figure 1:**
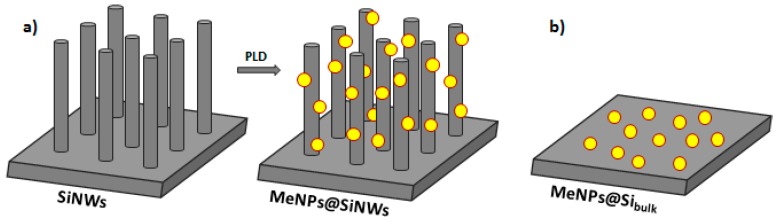
(**a**) Schematic of pulsed laser decoration of SiNWs (Me = Au or Cu) and (**b**) Metal NPs supported on bulk silicon.

**Figure 2:**
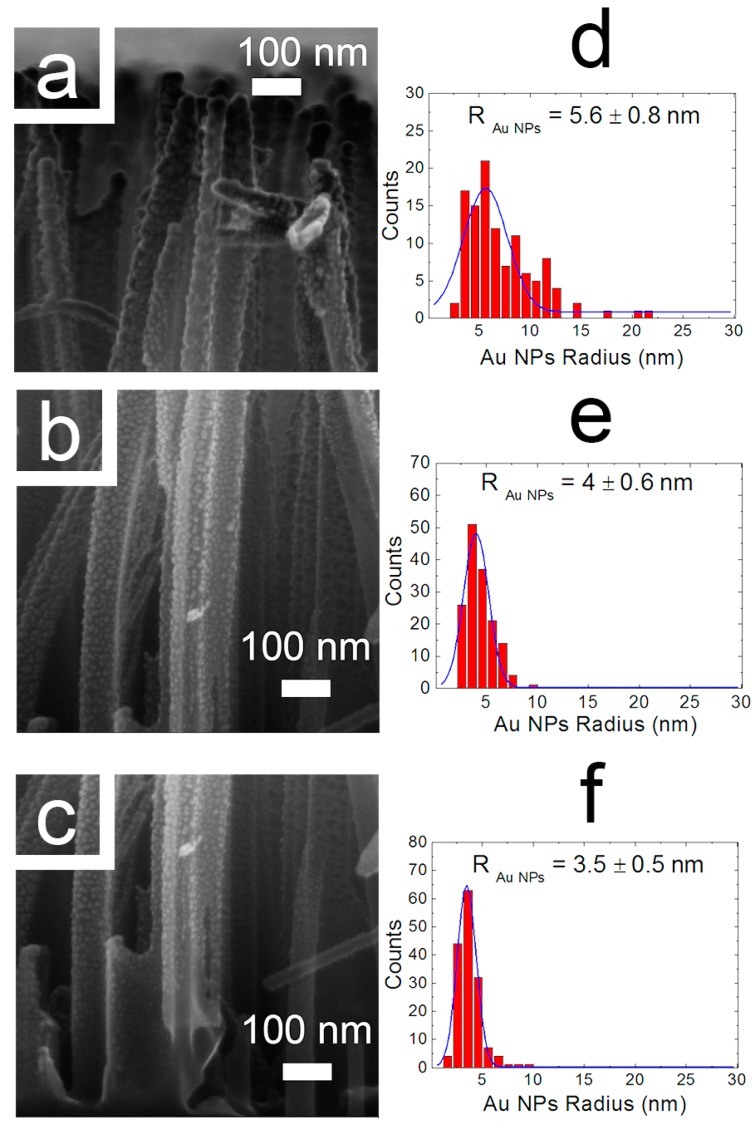
Cross-section SEM images (Field Emission Zeiss Supra 25 Microscope) of the (**a**) top, (**b**) center and (**c**) bottom regions of a 1.5 µm long SiNW array decorated with AuNPs. The statistical analysis of the AuNPs radius measured for the decorated sample are reported for the top (**d**), center (**e**) and bottom (**f**) sections, respectively.

**Figure 3:**
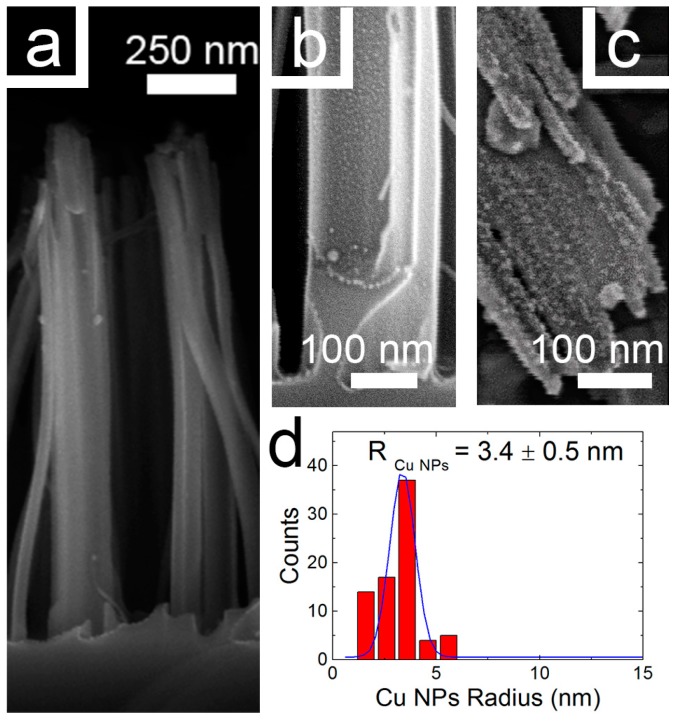
(**a**) Cross-section SEM image of Cu decorated SiNWs; (**b**) SEM microscopy displaying the NW bottom decorated with CuNPs; (**c**) SEM microscopy displaying the tips of an ensemble of Cu decorated SiNWs; (**d**) Statistical analysis of the CuNPs radius measured for the top section of the sample.

By adopting these conditions, a series of silicon wafers were decorated with gold and copper nanoparticles. Two nanocomposites based on Au and Cu NPs were prepared using the Si nanowires support (labeled as AuNPs@SiNWs and CuNPs@SiNWs, Figure 1a). For comparison, analogous Au and Cu nanocomposites were prepared by deposition on a bulk silicon wafer (labeled as AuNPs@Si_bulk_ and CuNPs@Si_bulk_, Figure 1b). The structural properties of these materials were investigated by scanning electron microscopy (SEM) as reported in Figure 2 and Figure 3, respectively. As already demonstrated [38], the SiNW arrays are not damaged during the PLD decoration process that was optimized in order to fully decorate the NW sidewalls. Figure 2 displays the SEM cross-section images of the (a) top, (b) center and (c) bottom regions of the Au decorated NWs.

The AuNPs radius distribution was measured for each section by software analysis and fitted by Gaussian distributions. The presence of a dense and coalesced distribution of bigger AuNPs is clearly attested in the top region with radius of 5.6 ± 0.8 nm (Figure 2), with size of 4 ± 0.6 nm in the center (2e) and 3.5 ± 0.5 nm in the bottom (2f) of the NWs. Figure 3a shows the SEM cross-section of SiNWs decorated with CuNPs. The presence of CuNPs along the whole NWs is visible from the top (3c) to the bottom (3b). In Figure 3b the SEM image of the NW bottom is reported. The uniform presence of CuNPs is visible but the size is at the detection limit of the SEM analysis. 

Figure 3c shows the SEM of the NW tips clearly demonstrating the presence of small NPs whose mean radius is of about 3.4 ± 0.5 nm (Figure 3d). This trend is in very good agreement with the results demonstrated for AgNPs [38] and AuNPs shown in Figure 2, in fact for CuNPs the radius distribution decreases along the NW from the top to the bottom. 

Dimensions of the NPs at the top of the NWs are close to the SEM resolution limit, therefore the NP size in the center and bottom region cannot be measured even if it is possible to appreciate their presence.

The PLD conditions adopted for Cu decoration led to the formation of NPs with smaller radii than Au ones, due to the different clustering dynamics during the PLD process and to the different wettability of the two metals with the SiNW substrate. 

RBS (Rutherford Backscattering Spectroscopy) analyses of CuNPs@SiNWs nanocomposite revealed the presence of 1.57 × 10^17^ Cu atoms/cm^2^ (corresponding to 1.67 × 10^−5^ g/cm^2^) and 1.95 × 10^17^ Au atoms/cm^2^ (6.41 × 10^−5^ g/cm^2^) for the analogous AuNPs@SiNWs material. 

AuNP- and CuNP-decorated SiNWs were also characterized by X-ray photoelectron spectroscopy (XPS) to provide information about their surface chemical composition and MeNP speciation. Typical surface composition of Au-based and Cu-based materials are reported in Tables S1 and S2 of the supplementary materials. Si% presents the lowest values for bulk materials (AuNPs@Si_bulk_ and CuNPs@Si_bulk_), due to the absence of substrate nanostructuration, which results in a sort of PLD-induced Si “metallization”. 

**Figure 4:**
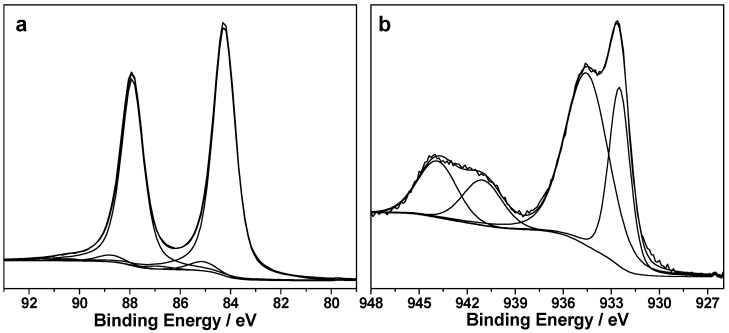
(**a**) Au4f XP region relevant to pristine AuNPs@SiNWs; (**b**) Cu2p_3/2_ XP region relevant to pristine CuNPs@SiNWs.

On the contrary, successful PLD decoration of SiNWs with nanosized metals was achieved, as indicated by the determined Au% and Cu%. Typical spectra relevant to Au4f and Cu2p_3/2_ regions obtained on pristine AuNPs@SiNWs and CuNPs@SiNWs materials, respectively, are presented in Figure 4. The main Au4f_7/2_ component falls at BE = 84.2 ± 0.1 eV (Figure 4a), which can be ascribed to gold coordinated to silicon in a silicide state in agreement with previous findings about energy shift towards higher BE when a Si–Au interface is created [41].

Other two Au4f_7/2_ components (at ~85 and 87 eV) represent less than 5% of the signal and can be attributed to oxidized gold species, within the limits related to their very low intensity. The Au4f spectrum of AuNPs@SiNWs is quite different from the one acquired for AuNP-decorated bulk Si, which is reported in Figure S1. In that case, it is present essentially a component at 83.8 ± 0.1 eV, compatible with bulk gold, and a second minor component at 84.2 ± 0.1 eV, whose explanation was already given. Cu2p_3/2_ region reveals the presence of two components, falling at *BE* = 932.4 ± 0.2 eV and 934.5 ± 0.2 eV (Figure 4b).

The former may be attributed to Cu(0)/Cu(I) species, however the analysis of CuL_3_M_45_M_45_ Auger region allows discriminating the two oxidation states (Figure S2).

The maximum of the Auger signal (at KE = 916.4 ± 0.2 eV) allows calculating the so-called modified Auger parameter (*α*′ = BE_Cu2p3_ + KE_CuLMM_), which is equal to 1848.8 ± 0.3 eV. This value is in agreement with the presence of Cu(I) [42].

The second component of the Cu2p_3/2_ photoelectron signal is undoubtedly ascribed to Cu(II), as also suggested by the occurrence of two shake-up features at BE > 940 eV [43]. It is not surprising that unstabilized CuNPs deposited on SiNWs are not at the elemental state: Cu(I) and Cu(II) species could be related to CuNP oxidation induced by air exposure. Finally, similar results about Cu speciation were found on Cu@Si bulk materials (Figure S3).

### 3.2. Catalytic Activity in C_aryl_–N Coupling Reactions

Bulk metallic copper barely shows catalytic activity, but it displays remarkable performances at the nanoscale level, as emerged in the recent developments of the copper(0) nanoparticle-catalyzed C–C and C-heteroatom bond formations and related reactions [44]. Likewise, gold nanoparticles based catalysts have been subjected to growing interest in recent times, with main applications in CO oxidation, reduction of nitrophenols, cycloadditions, hydrogen generation, Ullmann and Sonogashira couplings etc. [45,46].

Inspired by the recent literature [34], the C_aryl_–N coupling of aryl halides with amines (namely the Ullmann condensation) [47,48,49] was chosen as benchmark for SiNW supported MeNPs, a powerful tool for the synthesis of aromatic amines, which are valuable products possessing a variety of biological and pharmaceutical properties [50,51].

Although several homogeneous copper catalysts have been used for promoting this reaction (being Cu much cheaper than the other active metals in this process such as Pd and Rh) [52], only very few heterogeneous counterparts have been reported [34,53,54]. More importantly, no gold-based catalyst has been successfully employed until now in this kind of couplings.

**Figure 5:**
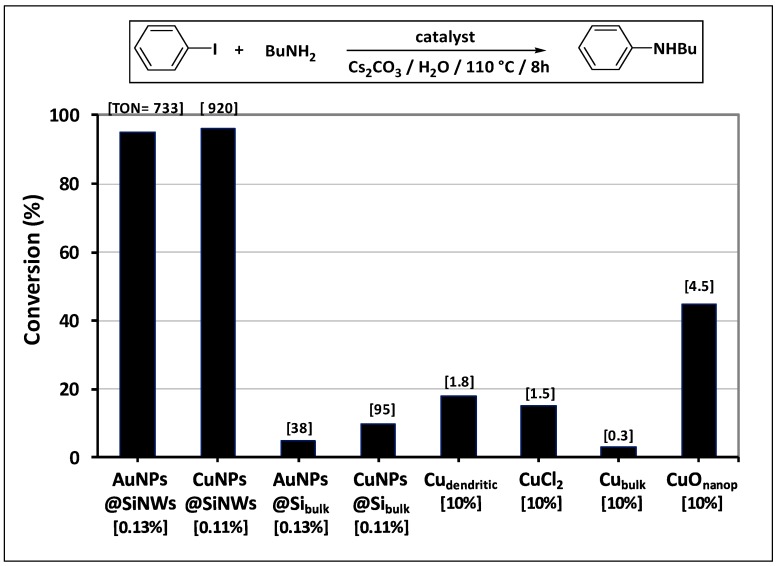
Comparison of catalytic performance in the C–N coupling of iodobenzene with butylamine (catalyst concentration is reported in square bracket). CuO_nanop._ = 33 nm in size (Sigma–Aldrich) [54], Cu_bulk_ = Cu powder (Sigma–Aldrich). Cu_dendritic_ = dendritic Cu (3 μm in size, Sigma–Aldrich) (for calculation of turn over number (TON) values see supplementary materials).

At first, catalytic performance of both Cu and Au nanocomposites were evaluated comparing their activity with that of similar catalysts in the literature (Figure 5). The coupling conditions were optimized on the model reaction between iodobenzene and butylamine, with preliminary experiments (Table S4) that showed Cesium carbonate and water as the most efficient base and solvent, respectively.

Under these conditions, unprecedented in the literature, both AuNPs@SiNWs and CuNPs@ SiNWs composites promoted the coupling at a very low catalyst concentration (0.11–0.13 mol %), with turn over number (TON) values of 733 and 920 (for Au and Cu materials, respectively), that are about ten up to hundred times higher than those of the most commonly used Cu catalysts (Figure 5). In contrast, the analogous nanocatalysts supported on the bulk silicon wafer (namely Au- and Cu-NPs@Si_bulk_) gave more modest results (TONs ten times lower), thus evidencing how the dense forest structure of Si nanowires is crucial for the catalytic efficiency (*vide infra*). 

Both the general applicability and limitations of this protocol were explored by means of the substrate scope showed in Table 1. From results clearly emerged that both Cu and Au based nanocatalysts show similar performances, with the highest activity displayed in the coupling between iodobenzene and primary aliphatic amines (yields in the range of 75–99%, Table 1, runs 1–8). Both steric and electronic factors affected the coupling in the predictable manner, with lower yields observed in the case of hindered substrates, such as dibutylamine and 2-iodotoluene (Table 1, runs 9 and 15), or with electron-rich substituents on the phenyl ring, like methoxy group (Table 1, runs 12–13).

As expected, the less nucleophilic aniline proved to be much less reactive as well as aryl bromides, that gave disappointing results (Table 1, runs 10, 16–17).

In contrast, a high reactivity was exhibited by the thiolic functionality, as demonstrated by the high efficiency with which 4-mercaptobutan-1-ol was converted into 4-(phenylthio)butan-1-ol (Table 1, runs 18). Conversely, alcoholic functionality proved to be unreactive (Table 1, run 19).

**Figure 6:**
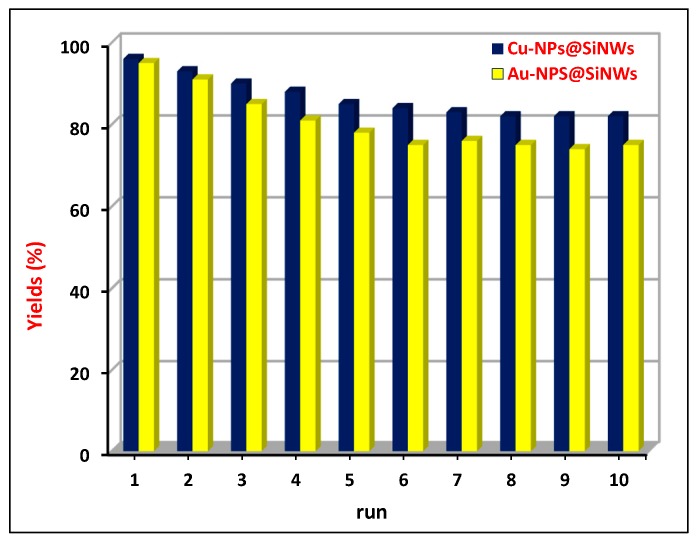
Recycling experiments for CuNPs@SiNWs and AuNPs@SiNWs catalysts in the C–N coupling of iodobenzene with butylamine.

The stability of both catalysts was studied in a series of recycling experiments. As shown in Figure 6, an initial decrease of activity occurred during the first four runs, certainly due to the depletion of metal nanoparticles in the upper part of nanowires because of the contact with bulk reaction solution. The depletion was testified by XPS analyses of hybrid materials after the first catalytic runs (see paragraph on mechanistic insight).

Next, the catalytic performances of both Au and Cu nanocatalysts remained unchanged in the range of 75–80% of yield throughout ten cycles, thus demonstrating the ability of SiNWs of anchoring metal NPs firmly within the interstitial spaces of wires impeding the further loss of catalytic material. The easy removal of the catalyst wafer from the reaction mixture (by tweezers) associated with the fact that catalysis is entirely heterogeneous in nature, as also confirmed by the hot filtration test (*vide infra*), enabled a recycle of the catalyst material much easier than that of other supported nanocatalysts that require tedious techniques such as filtration and ultracentrifugation.

### 3.3. Carbonylation of Haloarenes

Due to the successful results in C–N hetero-coupling, we decided to extend these findings to the carbonylation reaction. In the presence of proper nucleophiles, such as alcohols or amines, this transformation affords, through an analogous pathway initiated by the oxidative addition of metal to the haloarene, aromatic esters, and amides, which are important building blocks for various pharmaceutical and agrochemical compounds [55].

With very few exceptions, Iranpoor et al. recently reported the nickel-catalyzed version [56]—this process is dominated by palladium chemistry [57,58], but the extension to other versatile and much cheaper metals, such as copper, is of crucial importance for industrial applications. In addition, to the best of our knowledge, the use of copper and gold catalysts in the carbonylations of haloarenes has been totally neglected until now [44].

With this in mind, we carried out a series of preliminary experiments from which the copper-based material CuNPs@SiNWs emerged as the sole catalyst capable of promoting these reactions efficiently. The substrate scope showed an array of iodoarenes as suitable substrates that can be easily converted into the corresponding benzamides (or benzoates) in the presence of amines (or alcohols) under CO pressure (Table 2 and Table 3).

Slightly different reaction conditions were chosen for the two kinds of nucleophilic reagents. Amines were reacted in water at 110 °C for 8 h (at P_CO_ As expected, the less = 20 atm), while alcohols were used as reaction medium at 130 °C (P_CO_ As expected, the less = 30 atm), with Et_3_N as an additive. In the first case, water was a useful reaction medium capable of dissolving Cs_2_CO_3_ and suppressing, due to its poor nucleophilicity, the undesired side-reaction of solvolysis that leads to phenols.

Moreover, in the aminocarbonylation process small amounts of *N*-alkylanilines and α-ketophenyl acetamides by-products were also observed coming from the simple C–N coupling and the double carbonylation pathways, respectively. Notably, the latter side-reaction becomes predominant at CO pressures above 30 atm. Aliphatic amines were solely investigated obtaining high conversions (in the range of 75–97%) and selectivities (Table 2, runs 1–5). Predictably, both hindered and electron-rich haloarenes gave lower conversions (Table 2, runs 8–10), while bromobenzene proved to be unreactive (Table 2, run 11).

The excellent performance of supported Cu nanocatalyst were confirmed also in the alkoxycarbonylation of iodobenzene (Table 3). Reactions required Et_3_N as an additive and selectivities were almost always above 90%, being suppressed most of the above-mentioned side-reactions (Table 3, runs 1–5). Unfortunately, phenolic substrate was inert (Table 3, run 8). 

### 3.4. Reduction of Nitroarenes

Finally, to complete investigations on catalytic properties of these materials, the reduction of nitroaromatics was chosen as a further benchmark for evaluating their performances [59,60,61,62,63].

Nitrophenols and their derivatives are common water contaminants, that are present in wastewater effluents coming from the production of pesticides, difficult to eliminate. Alternatively, nitrophenol compounds could be reduced to aminophenol dyes and pharmaceutical products.

Removal with adsorption technique is one of the most effective methods, but it suffers from the generation of toxic slurries that remain technically or economically derivatives, which are useful in many applications that include photographic developers, corrosion inhibitors, dyeing agents, and the production of pharmaceutical products.

With this in mind, the catalyst efficiency of nanocomposites obtained in this study was compared with data for other catalysts in the reduction of 4-nitrophenol (Table 4). It should be noted that comparison among different catalysts is difficult since the activity of NPs depends on their size, shape, composition and concentration, but also on the reaction condition such as 4-nitrophenol and NaBH_4_ concentrations and temperature.

However, from data in Table 4 clearly emerged that our catalysts display performance comparable to or higher than the others, including the analogous composites prepared by galvanic decoration of silicon nanowires (Table 4, run 3).

**Figure 7:**
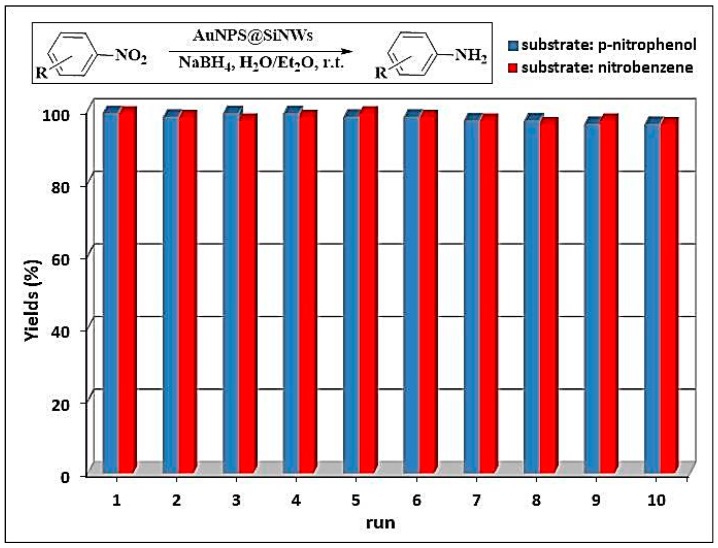
Recycling experiments for AuNPs@SiNWs catalyst in the reduction of nitroarenes with NaBH_4_.

The catalyst stability and reusability were assured in a number of consecutive cycles of reduction of 4-nitrophenol and nitrobenzene under the same reaction conditions. The results for gold catalyst indicate that nanocomposite could be successfully reused for 10 successive cycles without any loss of its activity (Figure 7). Similar results were also obtained in the case of analogous copper catalyst evidencing the high reusability of these supported nanoparticles. 

### 3.5. Mechanistic Insights

**Figure 8:**
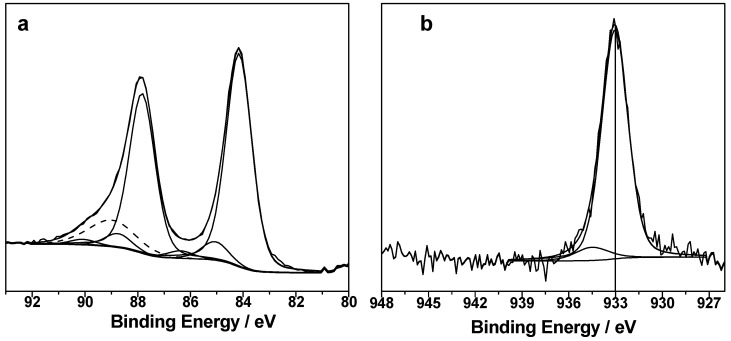
(**a**) Au4f XP region relevant to used AuNPs@SiNWs (the dashed line corresponds to Mg2s signal, due to contamination); (**b**) Cu2p_3/2_ XP region relevant to used CuNPs@SiNWs (the vertical line indicates the position of the main component).

Aiming at the investigation of the high catalytic activity shown by the hybrid nanomaterials herein proposed, we studied if and how the surface of the MeNP-decorated SiNWs was affected by a typical catalysis experiment. To this aim, XPS analysis was also carried out on MeNPs@SiNWs substrates after their use. Table S3 reports chemical composition of used AuNPs@SiNWs and CuNPs@SiNWs. It is evident that nanocatalyst depletion occurs, at least in the XPS sampling depth (10–20 nm). However, Au and Cu are still detectable and typical Au4f (a) and Cup_3/2_ (b) regions are reported in Figure 8. 

Regarding gold, the same components identified on pristine samples can be observed also on samples used in catalysis (Figure 8a). A clear prevalence of gold silicides is outlined. Additionally, a minor signal related to the Mg2s region (falling at about 89 eV) was identified, due to trace impurities of the chemicals used for reactions. The case of CuNPs is particularly interesting as a different main component falling at BE = 933.0 ± 0.2 eV is here identified (Figure 8b). This signal can be ascribed to Cu–Si bond, again a case of metal silicides, allowing these NPs to be in intimate contact with the SiNW surface.

Quite likely, the presence of Cu–Si interface was “masked” on pristine samples due to air oxidation and formation of surface oxides, which are preferentially sampled by XPS when they are located at the outer NP shell. 

On the contrary, copper silicide is clearly visible after nanocatalyst partial depletion. In fact, considering the systematic presence of metal silicides on the surface of samples used in catalysis, and the peculiar morphology of our hybrid catalytic system, a possible hypothesis of its unprecedented catalytic performance could be as follows.

We suppose that the high availability of MeNPs all over the length of NWs—as shown by microscopy data—allowed continuous recycling of the catalyst, as the voids among the wires looked like “micro-reactors”, confining the metal catalyst, which shows itself a great affinity towards nanostructured silicon. The latter entraps intermediate metal oxidation states (metal^(I)^ species) as silicides.

**Figure 9:**
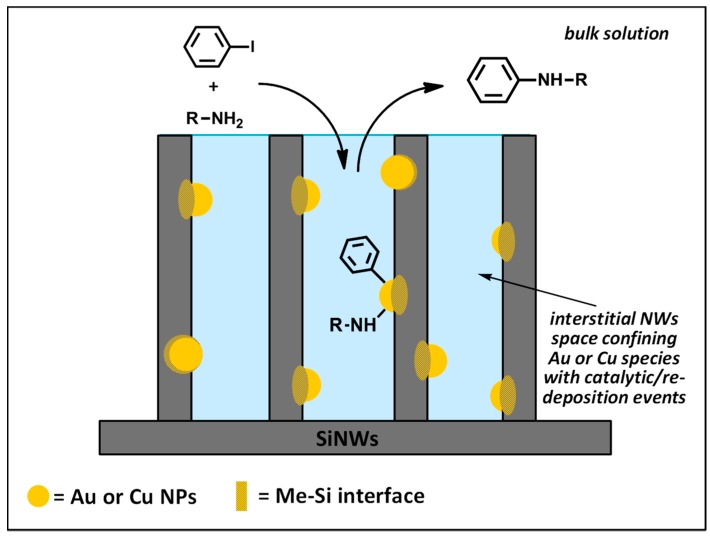
Schematic of C_aryl_–N coupling occurring into the interstitial space of SiNWs.

In this hypothesis, if catalytic metals undergo a series of reaction and re-deposition cycles within the micro-channels, this could amplify the catalytic properties of MeNPs, as schematized in Figure 9.

Besides chemical speciation of the active species by XPS technique, we obtained also precise indications on the heterogeneous nature of catalytic process from the studies of the solution activity after the separation of the silicon wafer catalyst (namely the hot filtration test). Thus, the model reaction between iodobenzene and butylamine was stopped after 3 h and the catalyst was removed with tweezers. The remaining liquid phase was re-heated for 5 h at 110 °C. After that time the GC analysis showed 37% conversion of iodobenzene, which corresponds to the yield of *N*-phenyl butylamine. The obtained yield was comparable to that obtained after 3 h (35%) and significantly lower than that noted after 8 h (96%, Table 2) of the catalytic reaction. 

**Figure 10:**
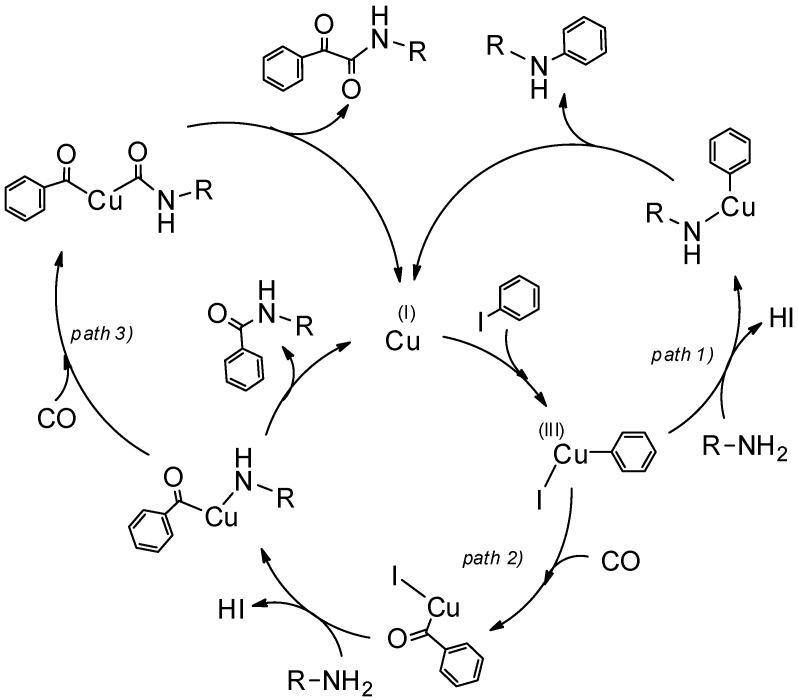
The plausible C–N coupling mechanism.

Considering the results of the XPS analyses, from which Cu(I) or Au(I) emerged as the dominant species on the NPs surface (mainly as “silicide”), taking into account the performed hot filtration tests and the composition of the obtained products, a plausible mechanism for the coupling of iodoarenes can be proposed (Figure 10).

The oxidative addition of Cu(I) to iodobenzene followed by nucleophilic attack of the amine onto the *σ*-aryl copper complex intermediate results in formation of the direct C_aryl_–N coupling product, *N*-alkylaniline (path 1).

Alternatively, under CO pressure, migratory insertion of CO leads to the *σ*-acyl copper intermediate (path 2), which evolves towards the aminocarbonylation product *N*-alkyl benzamide. At the presence of higher CO pressures, the *σ*-acyl copper intermediate experiences a further migratory insertion of CO leading to the dicarbonylation by-product, α-ketoamide (path 3). It can be assumed that in the case of gold based nanocomposite (AuNPs@SiNWs) pathways (2) and (3) are inibited due to the slow rate of CO migratory insertion step. Therefore, the direct C–N coupling (path 1) is solely observed.

## 4. Conclusions

In summary, silicon nanowires are well-known and powerful substrate supports for metal nanocatalysts. In this paper, we reported (for the first time) that the use of pulsed laser ablation technique for anchoring Cu- and Au-NPs on SiNWs enables these hybrid nanomaterials to act as superior heterogeneous catalysts. 

The advantages that can be reached are summarized below:

(i) an unexpected activity for some chemical transformations like C_aryl_–N couplings and subsequent carbonylations that have no precedents in the literature for gold and copper catalysts, respectively;
(ii) an extraordinary activity, attested by the very high TON values (about ten up to hundred times higher than those of the most commonly used catalysts (Figure 5)), surely due to the uniform coverage along the NW length and absence of the chemical shell surrounding the MeNPs;
(iii) a high recyclability, with more than ten recycling runs occurring with limited loss of activity, which can be ascribed to two main effects: (a) the high availability of MeNPs all over the length of NWs, notwithstanding the initial depletion in the upper layer of wires, and (b) the strong covalent interaction at the Me–Si interface by virtue of metal “silicides” formation. This confines the metal catalyst to the voids among the wires, transforming them into “micro-reactors” (Figure 9).

All these gains bring significant benefits in terms of efficiency and eco-sustainability to the investigated processes of C–N couplings and nitroarenes reductions. They also allow for easy fabrication of SiNW-supported metal nanoparticles with excellent characters, giving them potential use for many applications as multifunctional, high-performance catalysts.

## Figures and Tables

**Table 1:**
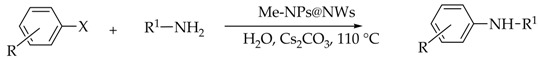
Substrate scope in the coupling of aryl halides and amines catalyzed by MeNPs@SiNWs ^(a)^.

Run	X	R	Amine (R^1^NH_2_)	Yield (%) ^(b)^	
				CuNPs@SiNWs	AuNPs@SiNWs
1	I	H	BuNH_2_	96	95
2	I	H	AllylNH_2_	80	85
3	I	H	BnNH_2_	99	98
4	I	H	HO(CH_2_)_4_NH_2_	75 ^(^^c)^	80 ^(^^c)^
5	I	H	H_2_N(CH_2_)_4_NH_2_	99 ^(^^d)^	95 ^(^^d)^
6	I	H	CyNH_2_	98	97
7	I	H	2-NH_2_CyNH_2_	99 ^(^^e)^	99 ^(^^e)^
8	I	H	n-C_12_H_25_NH_2_	82	79
9	I	I	Bu_2_NH	20	19
10	I	H	PhNH_2_	15	18
11	I	4-NO_2_	BuNH_2_	99	96
12	I	4-Br	BuNH_2_	50	55
13	I	4-MeO	BuNH_2_	40	49
14	I	4-CH_3_	BuNH_2_	94	98
15	I	2-CH_3_	BuNH_2_	72	73
16	Br	H	BuNH_2_	15	10
17	Br	4-CN	BuNH_2_	19	20
18	I	H	HO(CH_2_)_4_SH ^(f)^	90	95
19	I	H	HO(CH_2_)_4_OH	<1	3

^(a)^ General conditions: haloarene (0.25 mmol), amine (2.5 mmol), catalyst MeNPs@SiNWs (1.5 µm, 1 cm^2^), Cs_2_CO_3_ (1 mmol), H_2_O 2,0 mL, *T* = 110 °C, time 8 h; ^(b)^ Based on GLC areas using 4-methylanisole as an internal standard (selectivities were always higher than 95%). ^(c)^ 4-(phenylamino)butan-1-ol was the reaction product. ^(d)^ Only the monoarylation product was observed. ^(e)^ N^1^-phenylcyclohexane-1,2-diamine was the reaction product. ^(f)^ A mercaptane was used in place of amine: 4-(phenylthio)butan-1-ol was the reaction product.

**Table 2:**
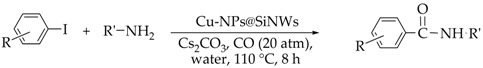
Aminocarbonylation of iodoarenes catalyzed by CuNP@SiNWs ^(a)^.

Run	R	R′-NH_2_	Product	Yields ^(^^b)^ (%)	Sel. ^(^^b)^ (%)
1	H	BuNH_2_	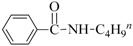	97	84
2	H	AllylNH_2_	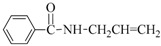	89	85
3	H	BnNH_2_	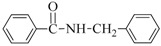	90	80
4	H^(c)^	4-AB	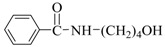	95	83
5	H	CyNH_2_	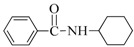	92	82
6	4-NO_2_	BuNH_2_	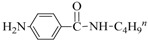	99	55 ^(d)^
7	4-Br	BuNH_2_	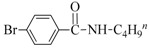	98	64
8	4-MeO	BuNH_2_	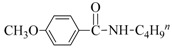	85	88
9	4-Me	BuNH_2_	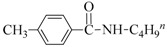	88	86
10	2-Me	BuNH_2_	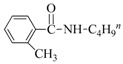	75	85
11	H ^(^^e)^	BuNH_2_	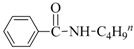	<5	-

^(a)^ Reaction conditions: haloarene 0.25 mmol, amine 0.625 mmol, Cs_2_CO_3_ 1 mmol, CuNP@SiNWs (1.5 µm, 1 cm^2^), H_2_O 2 mL, *T* = 110 °C, time = 8 h, P_CO_ = 20 atm. ^(b)^ Based on GC areas using anisole as an internal standard. ^(c)^ 4-AB= 4-aminobutan-1-ol. ^(d)^ 4-amino-*N*-butylbenzamide was the main reaction product. ^(e)^ Bromobenzene as substrate.

**Table 3:**
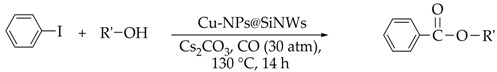
Alkoxycarbonylation of iodobenzene catalyzed by CuNP@SiNWs ^(a)^.

Run	R′-OH	Product	Yields ^(^^b)^ (%)	Sel. ^(^^b)^ (%)
1	Me-OH	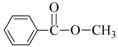	89	90
2	Bu-OH	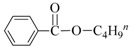	82	95
3	*^i^*Pr-OH	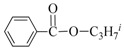	62	93
4	Allyl-OH	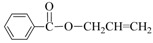	72	92
5	Cy-OH	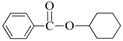	68	91
6	Bn-OH	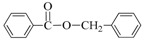	90	65
7	1,3-BD ^(^^c)^	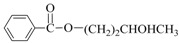	66	55
8	Ph-OH ^(^^c)^	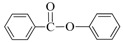	<5	-

^(a)^ Reaction conditions: iodobenzene 0.25 mmol, Cs_2_CO_3_ 1 mmol, cat. CuNPs@SiNW (1.5 µm 1 cm^2^), additive (Et_3_N) 1 mmol, alcohol 2 mL, *T* = 130 °C, time = 14 h, P_CO_= 30 atm; ^(b)^ Based on GC areas using anisole as an internal standard; ^(c)^ 1,3-BD = 1,3-butandiol. ^(^^d)^ Carried out with 2.5 mmols of phenol in 2 mL of acetonitrile.

**Table 4:**
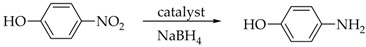
Comparison of catalyst performance in the reduction of p-nitrophenol ^(a)^.

Run	Catalyst	TOF ^(b)^	Ref.
1	AuNPs@SiNWs	123	This work
2	CuNPs@SiNWs	255	This work
3	CuNPs@SiNWs	220	[33]
5	Au/CeO_2_	240	[64]
6	Cu cubes	136	[65]

^(a)^ Reaction conditions for runs 1–2: p-nitrophenol 0.05 mmol (1 mL of 5·10^−2^ M water solution), NaBH_4_ 1 mmol (2.0 mL of 0.5 M water solution), cat. MeNPs@SiNWs wafer 0.5 cm^2^, H_2_O/Ether (2.5/0.85 mL), room temperature; ^(b)^ For TOF evaluation see supplementary materials.

## Supplementary Materials

The following are available online at http://www.mdpi.com/2079-4991/8/2/78/s1: XPS analyses, Optimization of C_aryl_–N coupling conditions, Evaluation of TON and TOF values. MS data of reaction products.

## References

[1] Ghosh R., Giri P.K. (2017). Silicon nanowire heterostructures for advanced energy and environmental applications: A review. Nanotechnology.

[2] Namdari P., Daraee H., Eatemadi A. (2016). Recent Advances in Silicon Nanowire Biosensors: Synthesis Methods, Properties, and Applications. Nanoscale Res. Lett..

[3] Liao F., Wang T., Shao M. (2015). Silicon nanowires: Applications in catalysis with distinctive surface property. J. Mater. Sci. Mater. Electron..

[4] Picca R.A., Calvano C.D., Lo Faro M.J., Fazio B., Trusso S., Ossi P.M., Neri F., D’Andrea C., Irrera A., Cioffi N. (2016). Functionalization of silicon nanowire arrays by silver nanoparticles for the laser desorption ionization mass spectrometry analysis of vegetable oils. J. Mass Spectrom..

[5] Irrera A., Leonardi A.A., Di Franco C., Lo Faro M.J., Palazzo G., D’Andrea C., Manoli K., Franzo G., Musumeci P., Fazio B. (2017). New Generation of Ultrasensitive Label-Free Optical Si Nanowire-Based Biosensors. ACS Photonics.

[6] Wang Y., Wang T., Da P., Xu M., Wu H., Zheng G. (2013). Silicon Nanowires for Biosensing, Energy Storage, and Conversion. Adv. Mater..

[7] Schmidt V., Wittemann J.V., Gösele U. (2010). Growth, Thermodynamics, and Electrical Properties of Silicon Nanowires. Chem. Rev..

[8] Wagner R.S., Ellis W.C. (1964). Vapor-Liquid-Solid Mechanism of Single Crystal Growth. Appl. Phys. Lett..

[9] Wu Y., Yang P. (2001). Direct Observation of Vapor-Liquid-Solid Nanowire Growth. J. Am. Chem. Soc..

[10] Swain B.S., Park J.-W., Yang S.-M., Mahmood K., Swain B.P., Lee J.-G., Hwang N.-M. (2015). Alignment of nanoparticles, nanorods, and nanowires during chemical vapor deposition of silicon. Appl. Phys. A Mater. Sci. Process..

[11] Morales A.M., Lieber C.M. (1998). A Laser Ablation Method for the Synthesis of Crystalline Semiconductor Nanowires. Science.

[12] Zhang R.Q., Lifshitz Y., Lee S.T. (2003). Oxide-Assisted Growth of Semiconducting Nanowires. Adv. Mater..

[13] Huang Z., Geyer N., Werner P., de Boor J., Gösele U. (2011). Metal-Assisted Chemical Etching of Silicon: A Review. Adv. Mater..

[14] Fazio B., Irrera A., Pirotta S., D’Andrea C., Del Sorbo S., Lo Faro M.J., Gucciardi P.G., Iatì M.A., Saija R., Patrini M. (2017). Coherent backscattering of Raman light. Nat. Photonics.

[15] Irrera A., Lo Faro M.J., D’Andrea C., Leonardi A.A., Artoni P., Fazio B., Picca R.A., Cioffi N., Trusso S., Franzo G. (2017). Light-emitting silicon nanowires obtained by metal-assisted chemical etching. Semicond. Sci. Technol..

[16] Toor F., Miller J.B., Davidson L.M., Nichols L., Duan W., Jura M.P., Yim J., Forziati J., Black M.R. (2016). Nanostructured silicon via metal assisted catalyzed etch (MACE): chemistry fundamentals and pattern engineering. Nanotechnology.

[17] Zhang M.L., Peng K.Q., Fan X., Jie J.S., Zhang R.Q., Lee S.T., Wong N.B. (2008). Preparation of Large-Area Uniform Silicon Nanowires Arrays through Metal-Assisted Chemical Etching. J. Phys. Chem. C.

[18] Elnathan R., Kwiat M., Patolsky F., Voelcker N.A.H. (2014). Engineering vertically aligned semiconductor nanowire arrays for applications in the life sciences. Nano Today.

[19] Galopin E., Barbillat J., Coffinier Y., Szunerits S., Patriarche G., Boukherroub R. (2009). Silicon Nanowires Coated with Silver Nanostructures as Ultrasensitive Interfaces for Surface-Enhanced Raman Spectroscopy. ACS Appl. Mater. Interfaces.

[20] Shao M.W., Zhang M.L., Wong N.B., Ma D.D., Wang H., Chen W., Lee S.T. (2008). Ag-modified silicon nanowires substrate for ultrasensitive surface-enhanced raman spectroscopy. Appl. Phys. Lett..

[21] Zhang M.L., Fan X., Zhou H.W., Shao M.W., Zapien J.A., Wong N.B., Lee S.T. (2010). A High-Efficiency Surface-Enhanced Raman Scattering Substrate Based on Silicon Nanowires Array Decorated with Silver Nanoparticles. J. Phys. Chem. C.

[22] Naama S., Hadjersi T., Menari H., Nezzal G., Ahmed L.B., Lamrani S. (2016). Enhancement of the tartrazine photodegradation by modification of silicon nanowires with metal nanoparticles. Mater. Res. Bull..

[23] Shao M., Wang H., Zhang M., Duo Duo Ma D., Lee S.-T. (2008). The mutual promotional effect of Au–Pd bimetallic nanoparticles on silicon nanowires: A study of preparation and catalytic activity. Appl. Phys. Lett..

[24] Amdouni S., Coffinier Y., Szunerits S., Zaïbi M.A., Oueslati M., Boukherroub R. (2016). Catalytic activity of silicon nanowires decorated with silver and copper nanoparticles. Semicond. Sci. Technol..

[25] Shao Y., Wei Y., Wang Z. (2011). Synthesis of silicon nanowires supported Ag nanoparticles and their catalytic activity in photo-degradation of Rhodamine B. Front. Optoelectron. China.

[26] Hua J., Shao M., Cheng L., Wang X., Fu Y., Ma D.D.D. (2009). The fabrication of silver-modified silicon nanowires and their excellent catalysis in the decomposition of fluorescein sodium. J. Phys. Chem. Solids.

[27] Yamada Y.M.A., Yuyama Y., Sato T., Fujikawa S., Uozumi Y. (2014). A Palladium-Nanoparticle and Silicon-Nanowire-Array Hybrid: A Platform for Catalytic Heterogeneous Reactions. Angew. Chem. Int. Ed..

[28] Liu L., Shao M., Wang X. (2011). Silicon Nanowires Supported Palladium Nanoparticles: An Efficient and Recyclable Heterogeneous Catalyst for Heck Reaction. Asian J. Chem..

[29] Wang F., Shao M., Cheng L., Chen D., Fu Y., Ma D.D.D. (2009). Si/Pd nanostructure with high catalytic activity in degradation of eosin Y. Mater. Res. Bull..

[30] Hu H., Shao M., Zhang W., Lu L., Wang H., Wang S. (2007). Synthesis of Layer-Deposited Silicon Nanowires, Modification with Pd Nanoparticles, and Their Excellent Catalytic Activity and Stability in the Reduction of Methylene Blue. J. Phys. Chem. C.

[31] Brahiti N., Hadjersi T., Menari H., Amirouche S., El Kechai O. (2015). Enhanced photocatalytic degradation of methylene blue by metal-modified silicon nanowires. Mater. Res. Bull..

[32] Qu Y., Zhong X., Li Y., Liao L., Huang Y., Duan X. (2010). Photocatalytic properties of porous silicon nanowires. J. Mater. Chem..

[33] Yang X., Zhong H., Zhu Y., Jiang H., Shen J., Huang J., Li C. (2014). Highly efficient reusable catalyst based on silicon nanowire arrays decorated with copper nanoparticles. J. Mater. Chem. A.

[34] Pan K., Ming H., Yu H., Huang H., Liu Y., Kang Z. (2012). Copper nanoparticles modified silicon nanowires with enhanced cross-coupling catalytic ability. Dalton Trans..

[35] D’Andrea C., Neri F., Ossi P.M., Santo N., Trusso S. (2009). The controlled pulsed laser deposition of Ag nanoparticle arrays for surface enhanced Raman scattering. Nanotechnology.

[36] Mikac L., Ivanda M., Gotic M., Maksimovic A., Trusso S., D’Andrea C., Foti A., Irrera A., Fazio B., Gucciardi P.G. (2015). Metal Nanoparticles Deposited on Porous Silicon Templates as Novel Substrates for SERS. Croat. Chem. Acta.

[37] Mollica Nardo V., Aliotta F., Mastelloni M.A., Ponterio R.C., Saija F., Trusso S., Vasi C.S. (2017). A Spectroscopic Approach to The Study of Organic Pigments In The Field of Cultural Heritage. Atti Accad. Pelorit. Pericol. Cl. Sci. Fis. Mat. Nat..

[38] D’Andrea C., Lo Faro M.J., Bertino G., Ossi P.M., Neri F., Trusso S., Musumeci P., Galli M., Cioffi N., Irrera A. (2016). Decoration of silicon nanowires with silver nanoparticles for ultrasensitive surface enhanced Raman scattering. Nanotechnology.

[39] Bailini A., Ossi P.M. (2007). Expansion of an ablation plume in a buffer gas and cluster growth. EPL Europhys. Lett..

[40] Spadaro M.C., Fazio E., Neri F., Trusso S., Ossi P.M. (2015). On the role of the ablated mass on the propagation of a laser-generated plasma in an ambient gas. EPL Europhys. Lett..

[41] Li Y., Shi W., Gupta A., Chopra N. (2015). Morphological evolution of gold nanoparticles on silicon nanowires and their plasmonics. RSC Adv..

[42] (2012). National Institute of Standards and Technology, “XPS Database”. http://srdata.nist.gov/xps.

[43] Cioffi N., Ditaranto N., Torsi L., Picca R.A., De Giglio E., Sabbatini L., Novello L., Tantillo G., Bleve-Zacheo T., Zambonin P.G. (2005). Synthesis, analytical characterization and bioactivity of Ag and Cu nanoparticles embedded in poly-vinyl-methyl-ketone films. Anal. Bioanal. Chem..

[44] Ranu B.C., Dey R., Chatterjee T., Ahammed S. (2012). Copper Nanoparticle-Catalyzed Carbon-Carbon and Carbon-Heteroatom Bond Formation with a Greener Perspective. ChemSusChem.

[45] Priecel P., Salami H.A., Padilla R.H., Zhong Z., Lopez-Sanchez J.A. (2016). Anisotropic gold nanoparticles: Preparation and applications in catalysis. Chin. J. Catal..

[46] Lukosi M., Zhu H., Dai S. (2016). Recent advances in gold-metal oxide core-shell nanoparticles: Synthesis, characterization, and their application for heterogeneous catalysis. Front. Chem. Sci. Eng..

[47] Ullmann F. (1903). Ueber Acridinsynthesen aus Aldehyden und aromatischen Basen. Ber. Dtsch. Chem. Ges..

[48] Hassan J., Sevignon M., Gozzi C., Schulz E., Lemaire M. (2002). Aryl−Aryl Bond Formation One Century after the Discovery of the Ullmann Reaction. Chem. Rev..

[49] Ley S.V., Thomas A.W. (2003). Modern Synthetic Methods for Copper-Mediated C(aryl)-O, C(aryl-N, and C(aryl)-S Bond Formation. Angew. Chem. Int. Ed..

[50] Annese C., Abbrescia D.I., Catucci L., Denora N., Fanizza I., Fusco C., Lapiana G. (2013). Site-dependent biological activity of valinomycin analogs bearing derivatizable hydroxyl sites. J. Pept. Sci..

[51] Annese C., D’Accolti L., Filardi R., Tommasi I., Fusco C. (2013). Oxidative cleavage of lactams in water using dioxiranes: An expedient and environmentally-safe route to ω-nitro acids. Tetrahedron Lett..

[52] Evano G., Blanchard N., Toumi M. (2008). Copper-Mediated Coupling Reactions and Their Applications in Natural Products and Designed Biomolecules. Synthesis. Chem. Rev..

[53] Rout L., Jammi S., Punniyamurthy T. (2007). Novel CuO Nanoparticle Catalyzed C−N Cross Coupling of Amines with Iodobenzene. Org. Lett..

[54] Jammi S., Sakthivel S., Rout L., Mukherjee T., Mandal S., Mitra R., Saha P., Punniyamurthy T. (2009). CuO Nanoparticles Catalyzed C−N, C−O, and C−S Cross-Coupling Reactions: Scope and Mechanism. J. Org. Chem..

[55] Brennführer A., Neumann H., Beller M. (2009). Palladium-Catalyzed Carbonylation Reactions of Aryl Halides and Related Compounds. Angew. Chem. Int. Ed..

[56] Iranpoor N., Firouzabadi H., Etemadi-Davan E., Nematollahi A., Firouzi H.R. (2015). A novel nickel-catalyzed synthesis of thioesters, esters and amides from aryl iodides in the presence of chromium hexacarbonyl. New J. Chem..

[57] Hajipour A.-R., Tavangar-Rizia Z., Iranpoor N. (2016). Palladium-catalyzed carbonylation of aryl halides: An efficient, heterogeneous and phosphine-free catalytic system for aminocarbonylation and alkoxycarbonylation employing Mo(CO)6 as a solid carbon monoxide source. RSC Adv..

[58] Prasada A.S., Satyanarayana B. (2013). Fe_3_O_4_ supported Pd(0) nanoparticles catalyzed alkoxycarbonylation of aryl halides. J. Mol. Catal. A Chem..

[59] Seo Y.S., Ahn E.-Y., Park J., Kim T.Y., Hong J.E., Kim K., Park Y., Park Y. (2017). Catalytic reduction of 4-nitrophenol with gold nanoparticles synthesized by caffeic acid. Nanoscale Res. Lett..

[60] Ciganda R., Li N., Deraedt C., Gatard S., Zhao P., Salmon L., Hernández R., Ruiza J., Astruc D. (2014). Gold nanoparticles as electron reservoir redox catalysts for 4-nitrophenol reduction: A strong stereoelectronic ligand influence. Chem. Commun..

[61] Zhang Z., Shao C., Zou P., Zhang P., Zhang M., Mu J., Guo Z., Li X., Wang C., Liu Y. (2011). In situ assembly of well-dispersed gold nanoparticles on electrospun silica nanotubes for catalytic reduction of 4-nitrophenol. Chem. Commun..

[62] Wang M.-L., Jiang T.-T., Lu Y., Liu H.-J., Chen Y. (2013). Gold nanoparticles immobilized in hyperbranched polyethylenimine modified polyacrylonitrile fiber as highly efficient and recyclable heterogeneous catalysts for the reduction of 4-nitrophenol. J. Mater. Chem. A.

[63] Tang R., Liao X.-P., Shi B. (2008). Heterogeneous Gold Nanoparticles Stabilized by Collagen and Their Application in Catalytic Reduction of 4-Nitrophenol. Chem. Lett..

[64] Liu B.C., Yu S.L., Wang Q., Hu W.T., Jing P., Liu Y., Jia W.J., Liu Y.X., Liu L.X., Zhang J. (2013). Hollow mesoporous ceria nanoreactors with enhanced activity and stability for catalytic application. Chem. Commun..

[65] Zhang P.H., Sui M.Y., Xiao G.J., Wang Y.N., Wang C.Z., Liu B.B., Zou G.T., Zou B. (2013). Facile fabrication of faceted copper nanocrystals with high catalytic activity for p-nitrophenol reduction. J. Mater. Chem. A.

