# Supplementary material for: Catalytic Activity of Silicon Nanowires Decorated with Gold and Copper Nanoparticles Deposited by Pulsed Laser Ablation
Source: Nanomaterials (Basel). 2018 Jan 30;8(2):78. doi: 10.3390/nano8020078 (PMC5853710; doi:10.3390/nano8020078)
Supplement: Supplementary file 1 [file nanomaterials-08-00078-s001.pdf]

Supplementary Materials

Catalytic activity of silicon nanowires decorated with gold and copper nanoparticles deposited by pulsed laser ablation

Michele Casiello,[1] Rosaria Anna Picca,[1] Caterina Fusco,[2] Lucia D'Accolti,[1,2] Antonio Alessio Leonardi,[3,4] Maria Josè Lo Faro,[3] Alessia Irrera,[3] Sebastiano Trusso,[3] Pietro Cotugno,[1] Maria Chiara Sportelli,[1] Nicola Cioffi,[1,*] Angelo Nacci,[1,2,*]

Contents:

XPS analyses (Tables S1-S3 and Figures S1-S3) .... page 2

Optimization of C_{aryl}-N coupling conditions (Table S4) .... page 5

Evaluation of TON and TOF values .... page 6

MS data of reaction products .... page 7

References .... page 12

XPS analyses

Typical surface composition and relevant regions for Au and Cu nanocomposites are reported below:

Table S1. Typical surface composition as determined by XPS analysis on AuNP-decorated materials, expressed in terms of element atomic%, error on gold percentage is ± 0.2%; error on the other elements is ± 0.5%

SAMPLE	C%	Au%	Si%	O%
**AuNPs@SiNWs**	44.2	40.6	3.6	11.6
**Au@Sibulk**	56.9	24.3	< 0.5	18.8

Table S2. Typical surface composition as determined by XPS analysis on CuNP-decorated materials, expressed in terms of element atomic%, error on copper percentage is ± 0.2%; error on the other elements is ± 0.5%

SAMPLE	C%	Cu%	Si%	O%
**CuNPs@SiNWs**	36.3	10.5	18.0	35.2
**Cu@Si bulk**	61.2	3.3	3.3	27.2

Table S3. Typical surface composition as determined by XPS analysis on used AuNP- and CuNP-decorated SiNWs, expressed in terms of element atomic%, error on gold and copper percentages is ± 0.2%; error on the other elements is ± 0.5%

SAMPLE	C%	Au% or Cu%	Si%	O%	K%	N%	Mg%
**Used AuNPs@SiNWs**	55.2	0.5	10.0	30.7	1.0	2.6	< 0.2
**Used CuNPs@SiNWs**	17.3	0.5	37.7	43.4	< 0.1	1.1	-

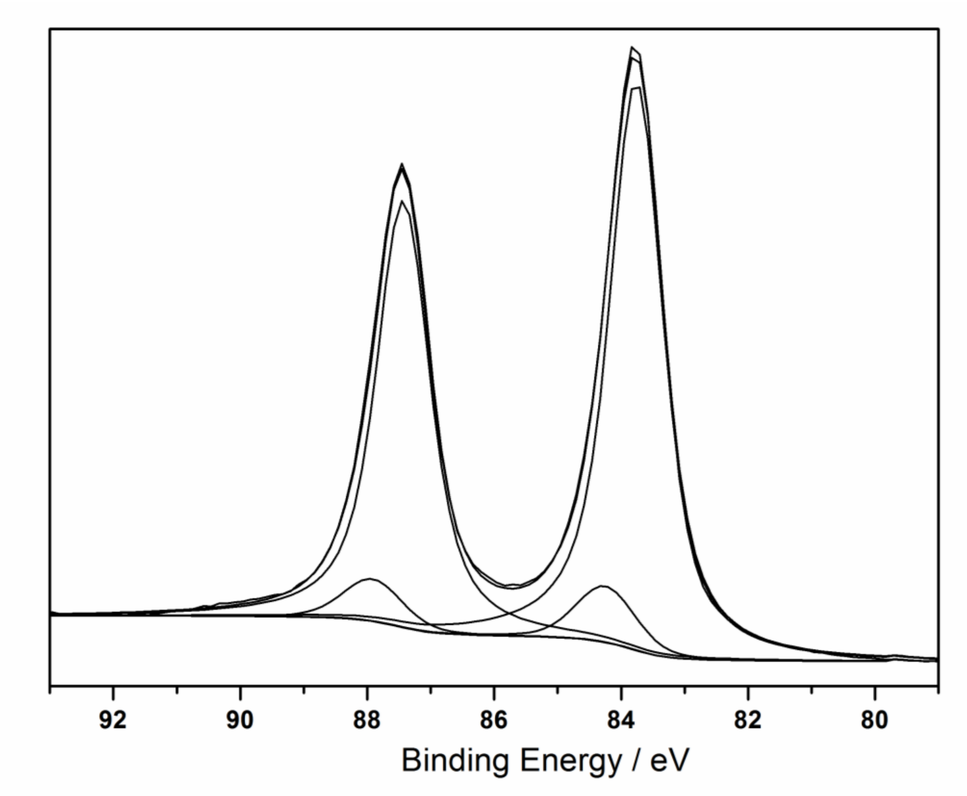

Figure S1. Au4f region relevant to Au@Si_{bulk} sample.

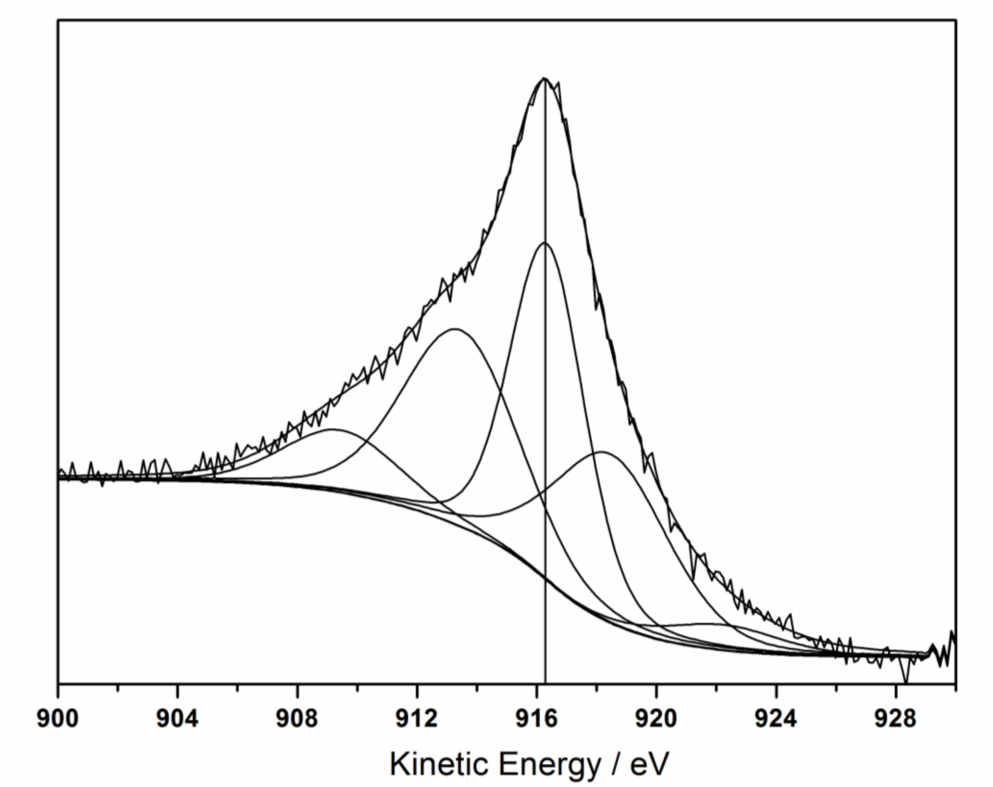

Figure S2. $CuL_3M_{45}M_{45}$ Auger region relevant to CuNPs@SiNWs. The line indicates the KE_{CuLMM} used to calculate α′.

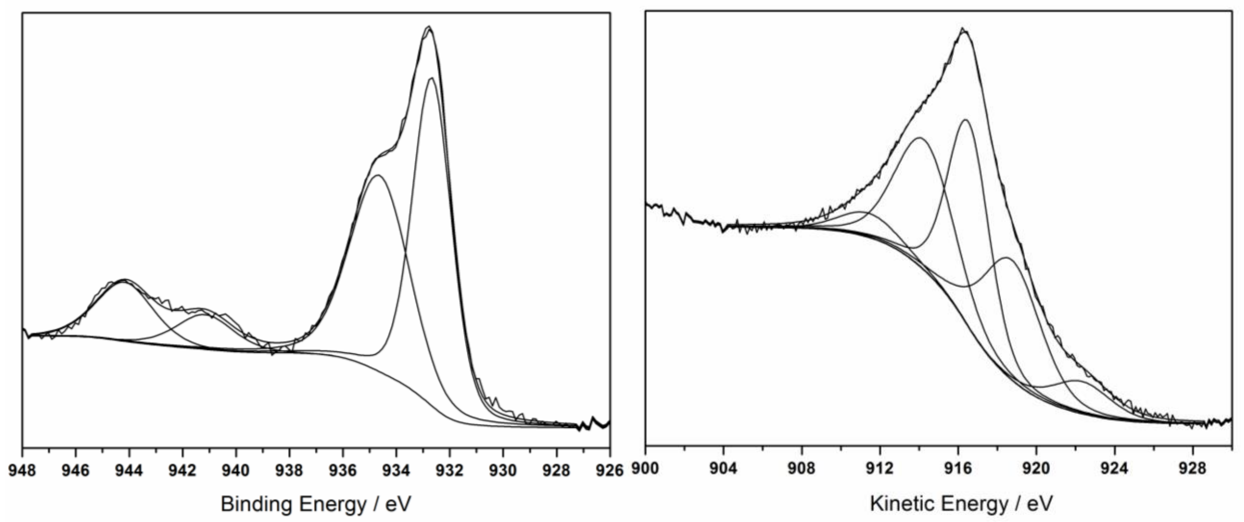

Figure S3. Cu2p$_{3/2}$ (left) and CuL$_3$M$_{45}$M$_{45}$ (right) regions relevant to CuNPs@Si$_{bulk}$.

Optimisation of coupling Caryl-N coupling conditions

Caryl-N coupling conditions were optimised on the model reaction between iodobenzene and butylamine promoted by CuNPs@SiNWs as the catalyst. Among the several bases investigated, Cesium carbonate provided the best performances (Table S4, runs 1-5); while water was the most efficient reaction medium most probably due to both the good solvent ability towards Cs_2CO_3 and the worse nucleophilic properties than amine that suppressed the competitive side-reaction of hydrolysis affording phenol (Table S4, runs 5-11).

Heating affected the coupling in a predicted manner: temperatures below 110 °C afforded modest yields, whereas higher temperature decreased selectivity (table S4, runs 12-13).

Table S4. Optimisation of C-N coupling conditions catalysed by Cu-NP@SiNWs.[a)]

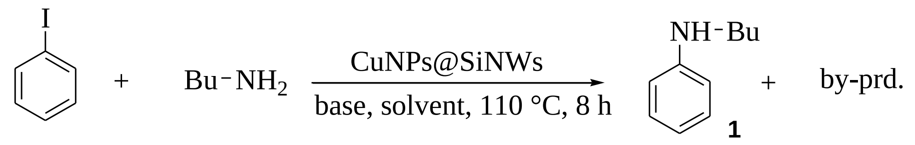

Run	Solvent	Base	Conv.[b)] (%)	Sel. (%)[b]	
				1	by-prd
1	H_2O	-	5	-	
2	H_2O	NEt_3	10	82	18
3	H_2O	Bu_4NOAc	15	66	34
4	H_2O	K_2CO_3	75	88	12
5	H_2O	Cs_2CO_3	98	96	4
6	CH_3CN	Cs_2CO_3	75	91	9
7	DMF	Cs_2CO_3	20	85	15
8	DMSO	Cs_2CO_3	10	-	
9	THF	Cs_2CO_3	20	78	22
10	DMA	Cs_2CO_3	<5	-	
11	H_2O/CH_3CN[c)]	Cs_2CO_3	40	95	5
12[d)]	H_2O	Cs_2CO_3	45	86	14
13[e)]	H_2O	Cs_2CO_3	100	52	48

[a)] Procedure as reported into the experimental section. General reaction conditions: Iodobenzene 0.25 mmol, Butylamine 0.625 mmol, Cs_2CO_3 1 mmol, solvent 2 mL, T= 110 °C, time= 8 hours. [b)] Based on GC areas. [c)] H_2O/CH_3CN = 3/1. [d)] T= 80 °C. [e)] T= 140 °C.

Evaluation of TON and TOF values

Both turnover number (TON) and turnover frequency (TOF) values were used to compare the catalytic activity of the nanocomposite with analogous catalysts reported in the literature (Fig. 5 and Table 4). TON is defined as the number of moles of converted iodobenzene per mole of surface Cu (or Au) atoms (Fig. 5), while TOF is the number of moles of converted p-nitrophenol per mole of surface Cu (or Au) atoms per hour (Table 4).[ref. 14a] Although only a small portion of surface metal atoms can actually serve as catalytic active sites, some of them will be bonded to silicon structure and unavailable for catalysis, it is common to take the total number of surface atoms as the number of active catalytic sites when the value is not known [ref. 65].

Hence, in the case of TON values of Fig. 5, calculations were carried out as follows:

Substrate: iodobenzene (0,25 mmol),<br>*Conversion:* 96%,<br>*Catalyst*: CuNPs@SiNWs (1.5 μm, 1 cm^2) [bearing 1.57 × 10^{17} Cu atoms/cm^2 (1.67 × 10^{-5} g/cm^2) which correspond to 2.61 × 10^{-4} mmol Cu/cm^2 (as resulted from Rutherford Backscattering Spectroscopy analyses).<br>$$\mathrm{TON}_{\mathrm{CuNPs@SiNWs}} = \frac{0.25 \times 0.96}{2.61 \times 10^{-4}} = 920$$	*Substrate*: iodobenzene (0,25 mmol),<br>*Conversion:* 95%,<br>*Catalyst*: AuNPs@SiNWs (1.5 μm, 1 cm^2) [bearing 1.95 × 10^{17} Au atoms/cm^2 (6.41 × 10^{-5} g/cm^2), which correspond to 3.24 × 10^{-4} mmol Au/cm^2 (as resulted from Rutherford Backscattering Spectroscopy analyses).<br>$$\mathrm{TON}_{\mathrm{AuNPs@SiNWs}} = \frac{0.25 \times 0.95}{3.24 \times 10^{-4}} = 736$$

In the case of TOF values reported in Table 4, calculations were carried in a similar manner:

Substrate: 4-nitrophenol (0,05 mmol),<br>*Conversion:* 100%,<br>Reaction time: 1.5 h<br>*Catalyst*: CuNPs@SiNWs (1.5 μm, 0.5 cm^2) which correspond to 1.31 × 10^{-4} mmolCu/cm^2<br>$$\mathrm{TOF}_{\mathrm{CuNPs@SiNWs}} = \frac{0.05}{1.31 \times 10^{-4} \times 1.5} = 255\ h^{-1}$$	*Substrate*: 4-nitrophenol (0,05 mmol),<br>*Conversion:* 100%,<br>Reaction time: 2.5 h<br>*Catalyst*: AuNPs@SiNWs (1.5 μm, 0.5 cm^2) which correspond to 1.62 × 10^{-4} mmolAu/cm^2<br>$$\mathrm{TOF}_{\mathrm{AuNPs@SiNWs}} = \frac{0.05}{1.62 \times 10^{-4} \times 2.5} = 123\ h^{-1}$$

MS data of reaction products

Reaction products were detected by GC–MS and identified by comparison of their MS spectra with the literature data. Below are reported both spectral experimental data and related literature:

<u>Caryl-N coupling products (table 1)</u>

N-butylaniline
MS m/z (%): 149 (12, M^+); 106 (100); 93 (3); 77 (14). Lit. [S1]

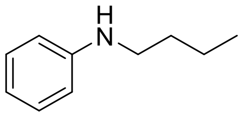

N-allylaniline
MS m/z (%): 133 (74, M^+); 117 (22); 106 (100); 92 (12); 77 (46). Lit. [S2]

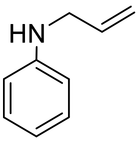

N-benzylaniline
MS m/z (%): 183 (40, M^+); 106 (18); 91 (100); 77 (20). Lit. [S3]

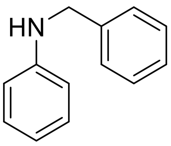

4-(phenylamino)butan-1-ol
MS m/z (%): 165 (11, M^+); 106 (100); 93 (5); 77 (17). Lit. [S4]

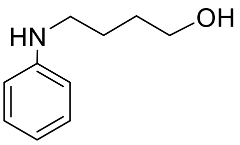

N-(4-aminobutyl)benzenamine
MS m/z (%): 164 (16, M^+); 147 (6); 119 (10); 106 (100); 93 (20); 77 (28). Lit. [S5]

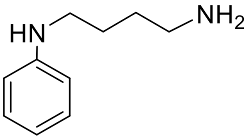

N-cyclohexylaniline
MS m/z (%): 175 (25, M^+); 132 (100); 118 (20); 106 (12); 93 (18); 77 (16). Lit. [S6]

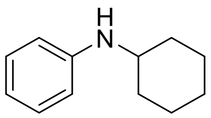

N-butyl-4-nitroaniline
MS m/z (%): 194 (20, M^+); 151 (100); 135 (5); 121 (5); 105 (71); 77 (6). Lit. [S6]

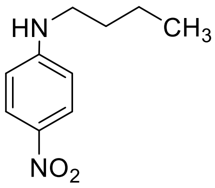

N-butyl-4-methoxyaniline
MS m/z (%): 179 (20, M^+); 116 (100); 121 (5); 108 (8); 77 (5). Lit. [S6]

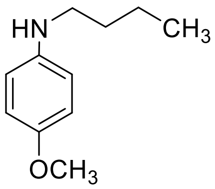

N¹-phenylcyclohexane-1,2-diamine
MS m/z (%): 190 (55, M^+); 172 (6); 147 (18); 132 (91); 118 (55); 106 (100); 97 (75); 77 (53). Lit. [S7]

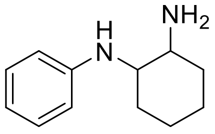

Diphenylamine
MS m/z (%): 169 (100, M^+); 167 (31); 115 (6); 83 (24); 77 (18). Lit. [S8]

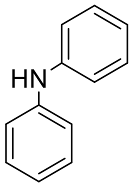

4-bromo-N-butylaniline
MS m/z (%): 228 (25, M^+); 184 (100); 157 (5); 118 (10); 105 (50); 91 (15); 77 (15). Lit. [S9]

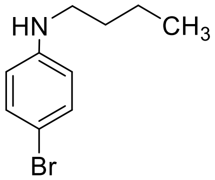

N-butyl-4-methylaniline
MS m/z (%): 163 (30, M^+); 120 (100); 106 (5); 91 (25); 77 (12). Lit. [S9]

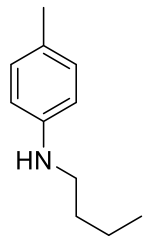

N-butyl-2-methylaniline
MS m/z (%): 163 (15, M^+); 120 (100); 106 (5); 91 (15); 77 (5). Lit. [S9]

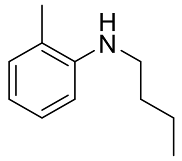

Aminocarbonylation products (table 2)

N-butylbenzamide
MS m/z (%): 177 (10, M^+); 135 (25); 105 (100); 77 (66). Lit. [S10]

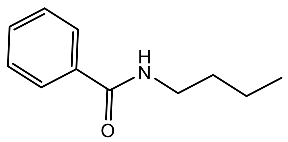

N-butyl-2-oxo-2-phenylacetamide
MS m/z (%): 205 (9, M^+); 105 (100); 77 (45); 57 (40). Lit. [S11]

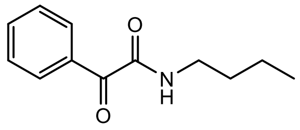

N-butyl-4-methylbenzamide
MS m/z (%): 191 (10, M^+); 148 (12); 119 (100); 91 (25). Lit. [S11]

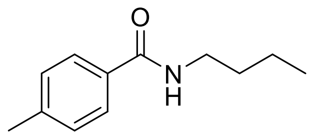

N-butyl-2-methylbenzamide
MS m/z (%): 191 (14, M^+); 148 (7); 176 (<5); 119 (100); 91 (50); 65 (25). Lit. [S11]

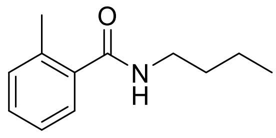

N-allylbenzamide
MS m/z (%): 161 (8, M^+); 146 (8); 105 (100); 94 (12); 77 (52). Lit. [S12]

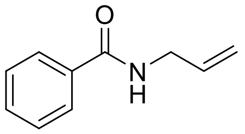

N-benzylbenzamide
MS m/z (%): 211 (50, M^+); 177 (10); 167 (5); 135 (10); 105 (100); 91 (18); 77 (60). Lit. [12]

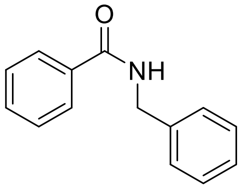

N-(4-hydroxybutyl)benzamide
MS m/z (%): 193 (10, M^+); 162 (5); 148 (15); 134 (10); 105 (100); 91 (18); 77 (50). Lit. [S13]

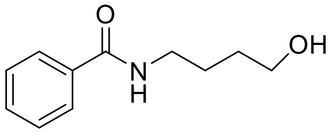

N-cyclohexylbenzamide
MS m/z (%): 203 (23, M^+); 160 (5); 122 (70); 105 (100); 77 (55). Lit. [S14]

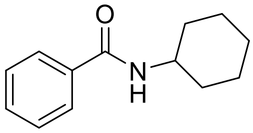

N-phenylbenzamide
MS m/z (%): 197 (30, M^+); 105 (100); 77 (60). Lit. [S14]

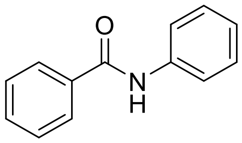

4-amino-N-butylbenzamide
MS m/z (%): 192 (12, M^+); 120 (100); 92 (20); 65 (22). Lit. [S15]

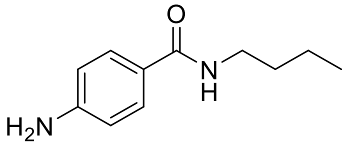

4-bromo-N-butylbenzamide
MS m/z (%): 257 (16, M^+); 255 (15, M^+); 213 (20); 185 (100); 157 (20); 104 (10); 76 (30). Lit. [S16]

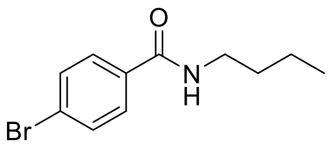

N-butyl-4-methoxybenzamide
MS m/z (%): 207 (15, M^+); 164 (15); 135 (100); 107 (10); 92 (14); 77 (18). Lit. [S17]

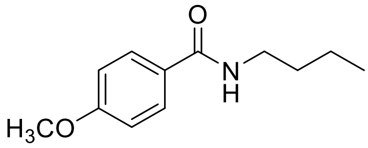

Alkoxycarbonylation products (table 3)

Methyl benzoatoe
MS m/z (%): 136 (27, M^+); 105 (100); 92 (5); 77 (65). Lit. [S12]

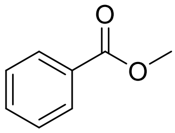

Isopropyl benzoate
MS m/z (%): 164 (7, M^+); 146 (5); 122 (34); 105 (100); 77 (45). Lit. [S12]

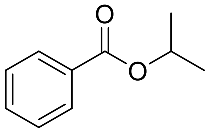

Butyl benzoate
MS m/z (%): 178 (<5, M^+); 123 (65); 105 (100); 91 (<5); 77 (65); 56 (25). Lit. [S18]

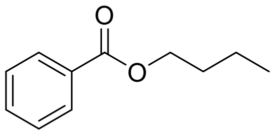

Allyl benzoate
MS m/z (%): 162 (5, M^+); 117 (4); 105 (100); 77 (47). Lit. [S19]

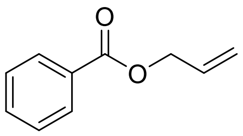

benzyl benzoate
MS m/z (%): 212 (10, M^+); 194 (5); 105 (100); 91 (70); 77 (50). Lit. [S14]

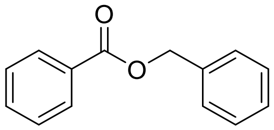

cyclohexyl benzoate

MS m/z (%): 204 (<5, M+); 123 (90); 105 (100); 77 (50); 67 (40). Lit. [S12]

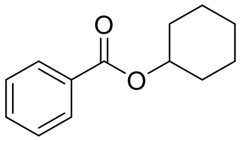

3-hydroxybutyl benzoate

MS m/z (%): 194 (<5, M+); 149 (10); 123 (40); 105 (100); 77 (60). Lit. [S20]

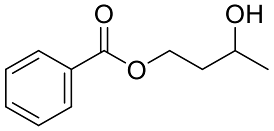

Reduction products (table 4 and Fig. 7)

4-aminophenol

MS m/z (%): 109 (100, M+); 96 (5); 80 (82); 77 (10). Lit. [S21]

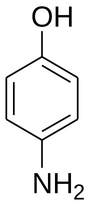

Aniline

MS m/z (%): 98 (100, M+); 66 (55). Lit. [S21]

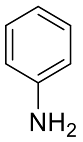

References

S1 Li, Y.-Q.; Chen, Y.-B.; Huang, Z.-Z. *Chinese Chemical Letters* **2014**, *25*, 1540–1544.

S2 Ohshima, T.; Miyamoto, Y.; Ipposhi, J.; Nakahara, Y.; Utsunomiya, M.; Mashima, K. *J. Am. Chem. Soc.* **2009**, *131*, 14317–14328.

S3 Zhang, G.; Scott, B. L.; Hanson, S. K. *Angew. Chem. Int. Ed.* **2012**, *51*, 12102 –12106.

S4 Compared with: https://www.chemsrc.com/en/Spectral/6517-80-2_1180315.html

S5 Ramirez, M. A.; Corona, M. V.; Blanco, M. M.; Perillo, I. A.; Porcal, W.; Salerno A. *Tetrahedron Lett* **2010**, *51*, 5000–5002

S6 Huang, L.; Yu, R.; Zhu, X.; Wan, Y. *Tetrahedron* **2013**, *69*, 8947-8977.

S7 Egli, v. M.; Hoesch, L.; Dreiding, A. S. *Helvetica Chimica Acta*, **1985** Vol. 68.

S8 Huang, L.; Yu, R.; Zhu, X.; Wan, Y. *Tetrahedron* **2013**, *69*, 8974-8977.

S9 Castillo, J.-C.; Orrego-Hernandez, J.; Portilla, J. *Eu. J. Org. Chem.* **2016**, 22, 3824–3835.

S10 Li, Y.; Chen, H.; Liu, J.; Wan, X.; Xu, Q. *Green Chem.*, **2016**, *18*, 4865-4870.

S11 Marosvolgyi-Hasko, D.; Kegl, T.; Kollar, L. *Tetrahedron* **2016**, *72*, 7509-7516.

S12 Mane, R. S.; Sasaki, T.; Bhanage, B. M. *RSC Adv.*, **2015**, *5*, 94776.

S13 Petersson, M. J.; Jenkins, I. D.; Loughlin, W. A. *Org. Biomol. Chem.*, **2009**, *7*, 739-746.

S14 Khedkart, M. V.; Sasaki, T.; Bhanage, B. M. *ACS Catal.* **2013**, *3*, 287-293.

S15 Wang, Y.; Wu, Z.; Li, Q.; Zhu, B.; Yu, L. *Catal. Sci. Technol.*, **2017**, *7*, 3747-3757.

S16 Shen, C.; Wei, Z.; Jiao, H.; Wu, X.-F. *Chem. Eur. J.* **2017**, *23*, 13369 –13378.

S17 Fang, T.; Gao, X.-H.; Tang, R.-Y.; Zhang, X.-G.; Deng, C.-L. *Chem. Commun.*, **2014**, *50*, 14775-14777.

S18 Gayakwad, E. M.; Patil, V. V.; Shankaarling, G. S. *New J. Chem.* **2017**, *41*, 2695-2701.

S19 Hemming, D. S.; Talbot, E. P.; Steel, P. G. *Tetrahedron lett.*, **2017**, *58*, 17-20.

S20 Hungerbühler, E.; Seebach, D.; Wasmuth, D. *Helvetica Chimica Acta* **1981**, *64*, 1467 – 1487.

S21 NIST Database and comparison with authentic sample.
